# Prevalence of perinatal post-traumatic stress disorder (PTSD) in low-income and middle-income countries: a systematic review and meta-analysis

**DOI:** 10.1136/bmjph-2023-000215

**Published:** 2024-05-02

**Authors:** Holly Jenkins, Zoe Daskalopoulou, Charles Opondo, Fiona Alderdice, Gracia Fellmeth

**Affiliations:** 1 Hertfordshire and West Essex Integrated Care Board, Welwyn Garden City, UK; 2 National Perinatal Epidemiology Unit, Nuffield Department of Population Health, University of Oxford, Oxford, UK; 3 Department of Medical Statistics, Faculty of Epidemiology and Population Health, London School of Hygiene and Tropical Medicine, London, UK; 4 School of Nursing and Midwifery, Queen's University Belfast, Belfast, UK

**Keywords:** public health, community health, social medicine

## Abstract

**Objectives:**

To systematically synthesise the evidence on prevalence of perinatal post-traumatic stress disorder (PTSD) in low-income and middle-income countries (LMICs).

**Design:**

Systematic review and meta-analysis.

**Data sources:**

MEDLINE, Embase, PsycINFO, Scopus, Web of Science, Global Health, Global Index Medicus and the grey literature were searched with no language or date restrictions. The final search was carried out on 3 May 2022.

**Eligibility criteria:**

Cross-sectional, cohort or case–control studies that assessed the prevalence of PTSD in pregnant or postpartum women in LMICs were included.

**Data extraction and synthesis:**

Screening, data extraction and quality assessment were conducted independently by two reviewers. Pooled prevalence estimates were calculated with 95% CIs and prediction intervals (PI) using random-effects meta-analyses. Subgroup analyses and meta-regression were conducted to explore possible sources of statistical heterogeneity.

**Results:**

39 studies were included in the systematic review of which 38 were included in meta-analysis. The pooled prevalence of clinically diagnosed perinatal PTSD was 4.2% (95% CI 2.2% to 6.8%; 95% PI 0–18%; 15 studies). The pooled prevalence of self-reported perinatal PTSD symptoms was 11.0% (95% CI 7.6% to 15.0%; 95% PI 0–36%; 23 studies). There was no evidence of differences in prevalence according to perinatal stage (antenatal versus postnatal), geographical region, type of setting or study quality.

**Conclusions:**

Findings of this review suggest 1 in 10 perinatal women experiences symptoms of PTSD and 1 in 20 experiences clinically diagnosed PTSD. Statistical heterogeneity between studies persisted in subgroup analyses and results should be interpreted with caution. More research from low-income countries is needed to improve understanding of the burden of perinatal PTSD in these settings.

**PROSPERO registration number:**

CRD42022325072.

**WHAT IS ALREADY KNOWN ON THIS TOPIC**

**WHAT THIS STUDY ADDS**

- Our systematic review suggests that in LMICs, approximately 1 in 10 perinatal women experience PTSD symptoms and 1 in 20 experience clinically diagnosed PTSD. Despite an increased focus on PTSD in LMICs in recent years, evidence from low-income (rather than middle-income) countries remains limited.

**HOW THIS STUDY MIGHT AFFECT RESEARCH, PRACTICE OR POLICY**

- This review highlights the importance of assessing perinatal women in LMICs for symptoms of PTSD in order to improve detection. Further research is required to determine the prevalence of PTSD in low-income countries and to explore the most feasible and cost-effective ways to identify and support women with perinatal PTSD.

## Introduction

 Mental disorders including depression, anxiety and post-traumatic stress disorder (PTSD) are among the most common morbidities of pregnancy and the postnatal period.^1^ Prevalence estimates of perinatal mental disorders vary significantly across diverse settings globally, with evidence suggesting that the risk of some conditions is higher in low-resource settings. Previous reviews have reported higher rates of perinatal depression and anxiety in low-income and middle-income countries (LMICs), compared with higher income countries.^2 3^ LMICs are defined by the World Bank on the basis of gross national income per capita, and the greater burden in these settings has been attributed to higher levels of maternal morbidity, poorer access to mental health services, greater exposure to psychosocial adversity including socioeconomic disadvantage, gender inequality, natural disasters and other chronic stressors.^4^,^6^ Although there has been an increase in studies of perinatal mental disorders from LMICs in recent years, the evidence remains skewed towards high-income settings. In particular, perinatal PTSD has received less attention than other mental disorders and the burden of perinatal PTSD in LMICs remains unknown.

To meet the diagnostic criteria for PTSD there must be a cluster of symptoms following exposure to a stressor event. The type of stressor is important, with evidence suggesting that highly traumatic events have a unique psychopathological impact compared with less dramatic life events.^7^ Symptoms include re-experiencing the traumatic event, avoidance of reminders, numbing of emotional responsiveness, negative alterations in mood and cognition and hyperarousal symptoms such as irritability and difficulty sleeping or concentrating.^8^ PTSD experienced during the perinatal period can be the result of pregnancy-related or birth-related factors (such as obstetric complications or a traumatic birth) or an exacerbation of pre-existing PTSD. Perinatal PTSD is associated with adverse health behaviours, negative impacts on relationships, higher risks of preterm birth, low birth weight and lower rates of breast feeding.^9^,^11^ The COVID-19 pandemic represented a unique stressor globally, with evidence suggesting that PTSD among perinatal women increased due to the exacerbation of known risk factors including relationship strains, increased levels of intimate partner violence and changes in antenatal care and birth experiences.^12 13^

Perinatal PTSD is a complex area of research and there are wide variations in prevalence estimates. One global review reported perinatal PTSD prevalence ranging from 0% to 40% across individual studies.^14^ These variations are attributable to a range of factors including inconsistencies in PTSD definitions and terminology as well as differences in population characteristics, timing of assessment and whether only birth-specific trauma or generalised traumatic events are included. While some studies use standardised self-report measures which assess for symptoms of PTSD, others use diagnostic interviews which provide a clinical diagnosis of PTSD. The distinction between these modes of assessment is crucial but often not clearly conveyed or differentiated in reported data.^15^ Previous reviews of perinatal PTSD have also not differentiated between LMICs and high-income countries (HICs). A review by Yildiz *et al* included publications from LMICs but did not report LMIC prevalence separately from the global pooled estimate.^14^ Our systematic review and meta-analysis addresses this important evidence gap by providing the first pooled prevalence estimate of perinatal PTSD in LMICs. We focus on general perinatal populations and report separate estimates for self-reported and clinically diagnosed PTSD in order to distinguish between symptomatology and clinical disorder.

## Methods

### Search strategy and inclusion criteria

A search strategy was developed using search terms relevant to perinatal status, PTSD and LMIC. The adapted School of Health and Related Research LMIC filter^16^ was applied. Search strategies were tailored to each database (online supplemental information SI1Supplementary ). The following electronic bibliographic databases were searched with no language or date restrictions: MEDLINE, Embase, PsycINFO, Scopus, Web of Science, Global Health and Global Index Medicus. The final search was carried out on 3 May 2022. To capture non-indexed publications and non-academic articles such as dissertations or reports, a grey literature search was conducted using *Google*, *Google Scholar* and African Journals Online (online supplemental information SI2). The first 20 pages of search results were screened with the assumption that beyond this there were unlikely to be further relevant results. Additionally, the websites of relevant organisations including the WHO were searched and reference lists of all included studies were manually searched. This systematic review is reported according to Preferred Reporting Items for Systematic Reviews and Meta-Analyses guidelines (online supplemental information SI3).^17^ The review protocol was registered on PROSPERO (CRD42022325072) on 25 April 2022.

Studies were included if they reported PTSD prevalence in general perinatal populations; were cross-sectional, cohort or case–control studies; focused on perinatal women in any trimester of pregnancy or up to 12 months post partum; used a diagnostic interview or validated self-report measure; and were conducted in LMICs as defined using World Bank country classifications. Validation studies were included if they met these criteria and reported prevalence from a diagnostic clinical interview. Conference abstracts were included if they met these criteria and all the data necessary for meta-analysis and quality assessment were reported. Intervention studies, systematic reviews, qualitative studies and editorials were excluded. We included studies of general perinatal samples which we defined as women attending general hospital or community-based maternity settings. Some women in these general perinatal samples would have experienced risk factors for PTSD such as pregnancy loss or exposure to natural disasters. However, studies were excluded if they focused *exclusively* on high-risk samples (eg, studies focusing exclusively on women who had experienced pregnancy loss; studies focusing specifically on women who had lived through a natural disaster).

### Screening, data extraction and quality assessment

Titles identified by searches were imported and deduplicated using Mendeley (V.2.68) and exported into Covidence where they were independently screened by two authors (HJ, ZD).^18^ Following abstract and title screening, full texts of potentially relevant articles were retrieved and assessed independently by two reviewers (HJ, ZD). Any disagreements were resolved through discussion with two other authors (FA, GF). Reasons for exclusion of full texts were documented. A data extraction form was developed and piloted. For each included article, data were extracted independently by two authors (HJ, ZD, GF) on setting, country, study design, age, timing of assessment, assessment instrument and prevalence. If studies reported only mean scores, did not report a cut-off point for clinically significant symptoms of PTSD or had other missing data, authors were contacted for additional information. The quality of included studies was assessed independently by two authors (HJ, ZD, GF) using an adapted version of the Joanna Briggs Institute (JBI) Checklist for Studies Reporting Prevalence Data (online supplemental information SI4).^19^ The JBI Checklist lists nine quality criteria, each answered as ‘yes’, ‘no’, ‘unclear’ or ‘not applicable’. Each study was categorised as being at low (eight or nine quality criteria met), moderate (six or seven quality criteria met) or high (five or fewer quality criteria met) risk of bias. Any discrepancies in methodological quality assessments were discussed with another author (FA).

### Data synthesis and analysis

Evidence from all included studies was summarised narratively. A meta-analysis was conducted using the number of cases and denominator from each study to calculate pooled prevalence of antenatal and postnatal PTSD with 95% CIs and 95% prediction intervals (PI) to illustrate which range of true prevalence rates might be expected across different settings.^20^ Random-effects analysis was used based on our assumption that each study estimates a different true underlying effect but that these effects follow a normal distribution which could be pooled.^21^ The random-effects model used the method of DerSimonian and Laird to estimate pooled prevalence, with the estimate of between-study heterogeneity being taken from the inverse-variance fixed-effect model. Freeman-Tukey double arcsine transformation was used to stabilise the variances. When data from the same participants were reported in more than one article (eg, multiple publications relating to the same study or cohort), only one article was selected for inclusion in the meta-analysis. The decision on which article to include in meta-analysis was based on the following criteria: prevalence from diagnostic clinical interview was prioritised over self-report measures, and larger sample sizes were prioritised over smaller sample sizes. When prevalence was reported for the same participants across multiple time points (eg, cohort studies that followed up participants through the perinatal period), data for all time points were extracted. However, to avoid double counting only one antenatal and one postnatal estimate were included in the main meta-analysis assessing antenatal versus postnatal pooled prevalence, and only one estimate from each study was included in subgroup analyses where a single pooled estimate was calculated for the perinatal period. When studies reported prevalence estimates using more than one cut-off, the cut-off was selected which was most comparable to other included studies unless authors provided justification for a different, locally validated cut-off.

The proportion of statistical heterogeneity attributable to between-study differences was assessed using the I^2^ statistic, with values >75% indicating considerable heterogeneity.^21 22^ Potential causes of heterogeneity were explored through subgroup analyses and meta-regression. These were planned a priori to assess the effects of timing of assessment (first, second and third trimesters of pregnancy; early (up to 1 month) vs late (1–12 months) post partum); setting (community vs hospital); mode of assessment (clinical diagnostic interview vs standardised self-report measures); geographical region (East Asia and Pacific; Europe and Central Asia; Latin America and Caribbean; Middle East and North Africa; South Asia; sub-Saharan Africa); country income level (middle-income vs low-income countries); study quality (high vs moderate vs low risk of bias); and timing in relation to the COVID-19 pandemic (data collection before vs after January 2020). As a significant difference in pooled prevalence was found between studies using self-report measures and those using diagnostic interviews, all other subgroup analyses were conducted separately for self-report studies and diagnostic studies. Due to insufficient data, it was not possible to conduct subgroup analyses by trimester of pregnancy, country income level, or pre-COVID-19 and post-COVID-19 pandemic. Publication bias was explored by visually assessing correlation between estimated prevalence and its SE. All analyses were conducted using *StataMP* (V.17).^23^

### Patient and public involvement

Patients or the public were not involved in the design, or conduct, or reporting or dissemination plans of our research.

## Results

**Figure 1:**
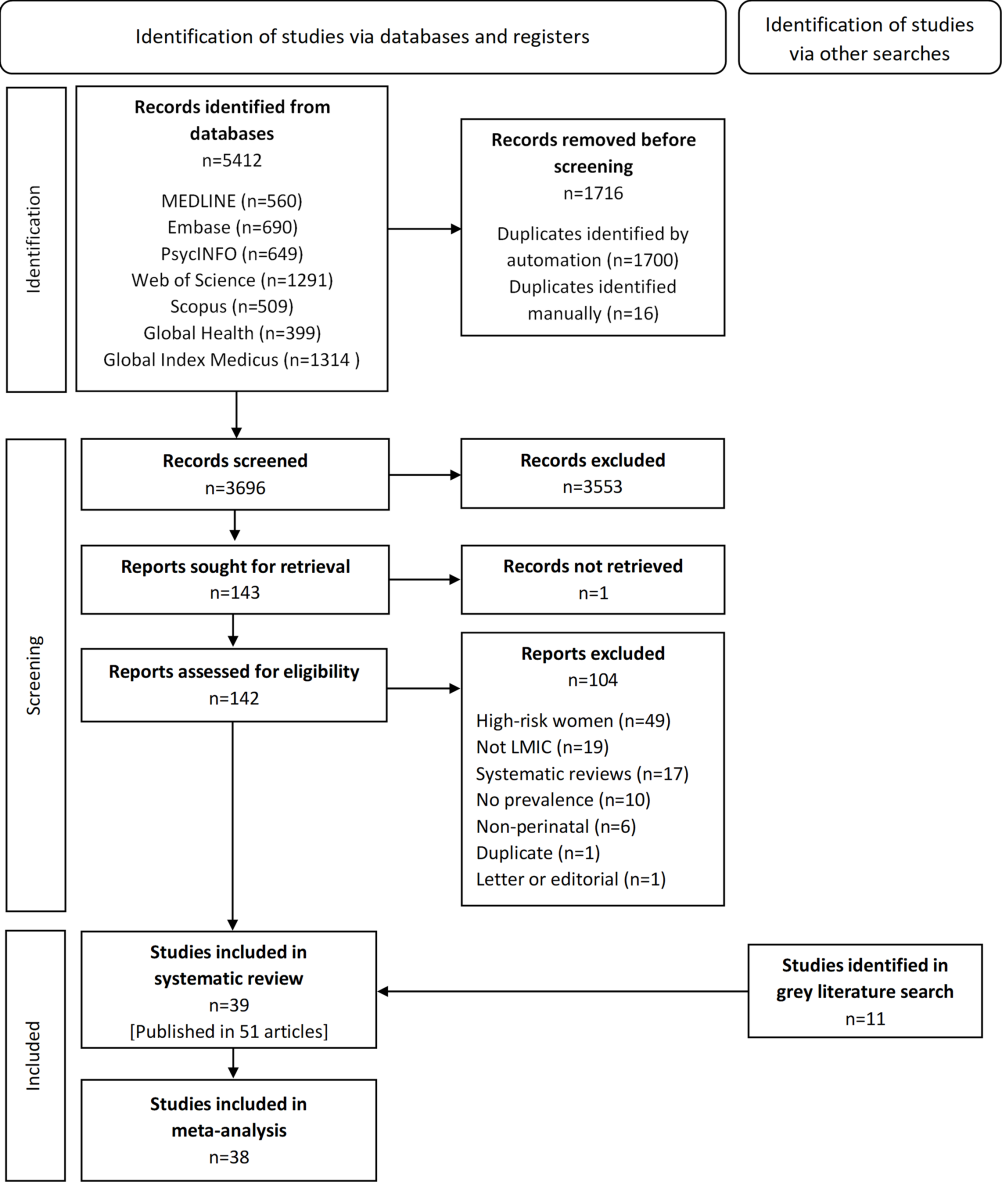
Preferred Reporting Items for Systematic Reviews and Meta-Analyses (PRISMA) flow diagram for study selection. LMIC, low-income and middle-income country.

Database searches identified 5412 articles of which 1716 were duplicates (figure 1). A total of 3696 titles and abstracts and 143 full texts were screened. 11 additional records were identified through grey literature searches. 14 authors were contacted for additional information of whom seven authors responded and six provided the required data. A total of 39 studies published in 51 articles were included in the systematic review,^24^,^73^ and of these 38^24^,^3032^ were included in meta-analysis. Reasons for exclusions are listed in figure 1.

### Study characteristics

Characteristics of the included studies are summarised in online supplemental information SI5. 15 studies assessed PTSD in the antenatal period, 21 studies assessed PTSD in the postnatal period and 3 studies assessed PTSD in both the antenatal and postnatal periods. Studies were predominantly cross-sectional (22 studies) or cohort (13 studies) studies; in addition, there were two validation studies^57 66^ and two case–control studies.^25 58^ 15 studies used clinical interviews to assess for the presence of PTSD: of these, 12 used the Mini International Neuropsychiatric Interview, 2 used the Structured Clinical Interview for the Diagnosis of DSM-IV (Diagnostic and Statistical Manual of Mental Disorders, Fourth Edition) Disorders and 1 used the Diagnostic Interview Schedule. The remaining studies used self-report measures to assess for the presence of symptoms of PTSD: these included the PTSD Checklist (PCL; 7 studies), PTSD Symptom Scale (5 studies); Perinatal PTSD Questionnaire (3 studies); and the Harvard Trauma Questionnaire (3 studies). Studies were conducted across 19 countries including Brazil (8 studies), India (3 studies), Nigeria (3 studies), South Africa (3 studies), Türkiye (3 studies), Iran (3 studies), China (2 studies), Timor-Leste (2 studies) and Tanzania (2 studies). Only two studies were conducted in low-income countries: Ethiopia and Liberia. The remaining studies were conducted in lower middle-income countries (17 studies) and upper middle-income countries (20 studies). The sample size of included studies ranged from 55^28^ to 2928.^59^ Most studies were conducted in hospitals (n*=*19) and community settings (n*=*16); two studies were conducted across both hospitals and the community and two conducted online. 17 studies were published after 2017. 37 studies were published in English, 1 in Portuguese^27^ and 1 in Chinese.^39^ Across individual studies, the reported prevalence of antenatal PTSD ranged from 0%^34 63^ to 40.7%^29^ and the reported prevalence of postnatal PTSD ranged from 0%^26 46 47^ to 52.9%.^69^ Quality assessment scores for each study are summarised in online supplemental information SI6. The risk of bias was rated as high, moderate and low in 15, 12 and 12 studies, respectively. The majority of studies used a sampling frame representative of the general perinatal population, applied appropriate inclusion and exclusion criteria and had adequate (>60%) response rates. Sample size calculations were frequently not reported.

### Prevalence of PTSD

**Figure 2:**
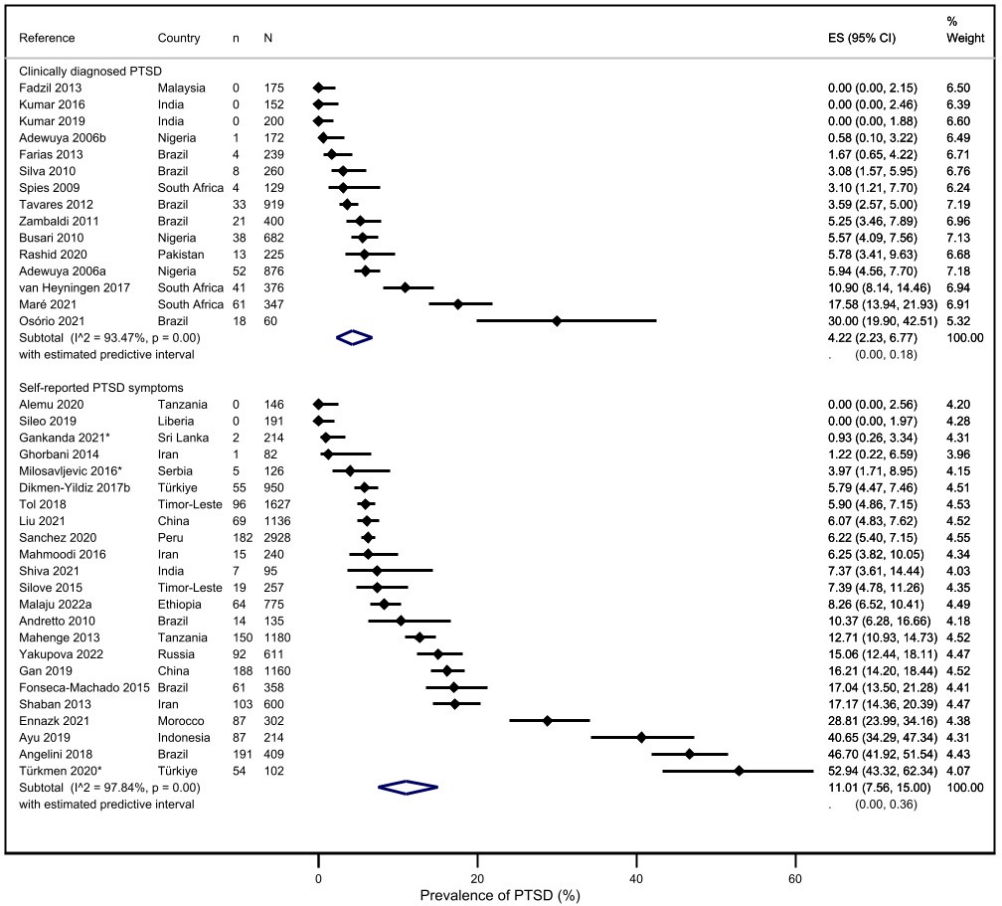
Prevalence of clinically diagnosed post-traumatic stress disorder (PTSD) and self-reported PTSD symptoms.

The pooled prevalence of clinically diagnosed PTSD across the perinatal period was 4.2% (95% CI 2.2% to 6.8%; 95% PI 0–18%; 15 studies).^2425 30 34 35 46 47 55 57 58 65^,^67 70 73^ The pooled prevalence of self-reported PTSD symptoms across the perinatal period was 11.0% (95% CI 7.6% to 15.0%; 95% PI 0–36%; 23 studies),^26^,^2932 33 37 39 40 42 48 50 52 53 56 60 62^ suggesting a statistically significantly (p=0.002) higher prevalence of self-reported PTSD symptoms compared with clinically diagnosed PTSD disorder (figure 2).

When antenatal and postnatal studies were assessed separately, the prevalence of clinically diagnosed PTSD was 4.1% (95% CI 1.2% to 8.3%; 95% PI 0–25%; 8 studies) antenatally^25 30 34 35 55 65 66 70^ and 5.4% (95% CI 2.3% to 9.8%; 95% PI 0–26%; 8 studies) postnatally.^24 46 47 55 57 58 67 73^ The prevalence of self-reported PTSD symptoms was 9.6% antenatally (95% CI 5.6% to 14.6%; 95% PI 0–31%; I^2^=97.5%; 8 studies)^29 32 37 50 63 64 68 74^ and 11.6% postnatally (95% CI 7.1% to 17.0%; 95% PI 0–41%; I^2^=97.6%; 17 studies).^26^,^2832 33 39 40 42 48 52 53 56 60 62 64 69 71^

### Subgroup analyses

Results of subgroup analyses are summarised in table 1 and forest plots are presented in online supplemental information SI7. For self-reported symptoms, prevalence was higher in the early postnatal period compared with the late postnatal period (20.2% vs 10.6%), while for clinically diagnosed PTSD, prevalence was lower in the early postnatal period compared with the late postnatal period (0.9% vs 7.3%), though these differences were not statistically significant. Prevalence was lowest in the South Asia region (self-reported symptoms 2.2%; 95% CI 0.8% to 4.3%; 2 studies; clinically diagnosed PTSD 0.9%; 95% CI 0.0% to 5.9%; 3 studies). Self-reported symptoms were highest in Latin America (18.1%; 95% CI 3.7% to 40.1%; 4 studies) and clinically diagnosed PTSD was highest in sub-Saharan Africa (6.4%; 95% CI 3.1% to 10.8%; 6 studies). None of the differences between regions reached statistical significance. No significant differences in prevalence estimates were seen between community-based and hospital-based studies or by study quality. Pooled estimates did not differ significantly according to risk of bias, though studies with low risk of bias had narrower 95% CIs than studies with high risk of bias. The small number of studies included in some subgroup analyses means results should be interpreted with caution. Results of fixed-effects meta-analysis were similar to those from random-effects models (online supplemental information SI8). Random-effects meta-regression analyses for subgroups suggested heterogeneity in pooled prevalence of clinically diagnosed PTSD by geographical region (p=0.03). However, there was little evidence of heterogeneity in pooled estimate by perinatal or postnatal stage, setting or study quality (online supplemental information SI9). The distribution of standardised prevalence estimates across studies was not asymmetrical, therefore providing no evidence for the presence of any publication bias (online supplemental information SI10).

**Table 1:** Pooled prevalence of PTSD by perinatal stage, geographical region, setting and study quality

	Self-reported PTSD symptoms				Clinically diagnosed PTSD			
	Studies (sample)	Pooled prevalence (95% CI)	95% PI(%)	I^2^(%)	Studies (sample)	Pooled prevalence (95% CI)	95% PI(%)	I^2^(%)
Perinatal stage								
Antenatal	8 (7705)	9.6 (5.6 to 14.6)	0–31	97.5	8 (2380)	4.1 (1.2 to 8.3)	0–25	94.4
Postnatal	17 (7253)	11.6 (7.1 to 17.0)	0–41	97.6	8 (3384)	5.4 (2.3 to 9.8)	0–26	95.1
Postnatal stage								
Early (0–1 month)	3 (453)	20.2 (0.2 to 58.6)	–	–	3 (577)	0.9 (0.0 to 5.9)	–	–
Late (1–12 months)	14 (6245)	10.6 (5.8 to 16.7)	0–42	97.8	3 (2347)	7.3 (2.8 to 13.8)	–	–
Geographical region*								
East Asia and Pacific	5 (4394)	13.2 (6.4 to 22.1)	0–54	98.2	1 (175)	–	–	–
Europe and Central Asia	4 (1789)	16.0 (5.1 to 31.3)	0–93	97.9	0	–	–	–
Latin America	4 (3830)	18.1 (3.7 to 40.1)	0–100	99.2	5 (1878)	5.7 (2.4 to 10.1)	0–27	90.4
Middle East and North Africa	4 (1224)	11.6 (3.6 to 23.3)	0–79	96.2	0	–	–	–
South Asia	2 (309)	2.2 (0.8 to 4.3)	–	–	3 (577)	0.9 (0.0 to 5.9)	–	–
Sub-Saharan Africa	4 (2292)	3.3 (0.1 to 10.2)	0–58	97.3	6 (2582)	6.4 (3.1 to 10.8)	0–26	93.3
Setting*								
Community	7 (3296)	11.7 (4.4 to 21.9)	0–56	98.1	8 (3341)	4.2 (1.7 to 7.8)	0–22	94.5
Hospital	14 (9836)	10.6 (6.4 to 15.8)	0–37	98.1	5 (1129)	2.0 (0.1 to 5.5)	0–23	89.7
Risk of bias*								
Low	8 (6797)	8.0 (5.4 to 11.0)	1–21	93.4	3 (1273)	3.8 (1.1 to 7.9)	–	–
Moderate	8 (3984)	3.2 (4.9 to 24.7)	0–63	98.8	3 (790)	3.4 (0.5 to 8.8)	0–44	94.2
High	7 (3057)	12.5 (4.9 to 22.7)	0–57	97.7	8 (2467)	5.0 (1.5 to 10.1)	0–30	95.3

* Antenatal estimatesand postnatal estimates combined.

PIprediction intervalPTSDpost-traumatic stress disorder

## Discussion

The pooled prevalence estimate for perinatal PTSD in LMIC settings was 4.2% for clinically diagnosed PTSD and 11.0% for self-reported symptoms of PTSD. These findings suggest that perinatal PTSD represents a significant burden within LMIC settings, with approximately 1 in 10 perinatal women experiencing PTSD symptoms and 1 in 20 perinatal women having a clinical diagnosis of PTSD. The prevalence of PTSD was similar in the antenatal and postnatal periods: the prevalence of clinically diagnosed PTSD was 4.1% antenatally and 5.4% postnatally, and the prevalence of self-reported symptoms of PTSD was 9.6% antenatally and 11.6% postnatally. However, high levels of statistical heterogeneity in the meta-analyses means these pooled estimates must be interpreted with caution.

Our estimates are not directly comparable with those of Yildiz *et al*^14^ due to differences in inclusion criteria and synthesis of results. Yildiz *et al* report a prevalence of PTSD using diagnostic measures (defined in their review as clinical interviews or self-report measures that use DSM diagnostic criteria) among community-based samples from predominantly HICs of 3.3% antenatally and 4.0% post partum: these estimates are in line with our estimates of clinically diagnosed PTSD of 4.1% and 5.4%, respectively.

The difference in estimates of clinically diagnosed PTSD compared with self-reported PTSD symptoms is unsurprising given that not all those with self-reported symptoms will meet the criteria for a clinical diagnosis. The magnitude of the difference (4.2% vs 11.0%) highlights the importance of distinguishing between the two—a distinction that is not always clear in reported estimates. Antenatal and postnatal prevalence estimates were similar, reflecting findings of a previous review.^14^ There was some evidence of differences in pooled prevalence across geographical regions, with lower prevalence in South Asia compared with other regions. Authors of a recent review of adolescent perinatal mental health in South Asia and sub-Saharan Africa called for more research to understand local conceptualisations of mental health and trauma.^75^ Studies from the East and Central Asia and Africa were limited and all but one of the studies from Latin America were conducted in Brazil, with differences in estimates seen even among studies from this single country.

Statistical heterogeneity persisted in subgroup analyses, and individual studies reported a wide variation in prevalence estimates ranging from 0% to over 50%. This variation may be explained by a number of possible factors. First, different self-report measures were used across studies, and although all measures were reported as having been validated, there may have been variation in the quality of translation and cultural adaptation of measures across studies. There is important emerging evidence of cross-cultural variability in the salience of avoidance or numbing symptoms and in the prevalence of somatic symptoms, suggesting that that ‘Western-centric’ diagnostic criteria may be less effective in detecting PTSD in LMIC contexts.^76^ Robust local validation of measures is therefore crucial as to avoid underdetection of PTSD. Second, the mode of administration of assessments—for example, whether self-report measures are read out loud by a healthcare worker or study team member or self-completed by respondents—may influence reported prevalence. Third, characteristics of study populations may have differed across studies. For example, studies which recruited participants from hospital settings may have included higher risk individuals than those recruiting from community-based settings. Finally, the use of LMIC as a category has received criticism for being overly broad and perpetuating unwarranted divisions between countries.^77^ The 163 countries currently classified as LMIC are highly diverse in terms of their populations, cultures and health infrastructure, and the variation in prevalence across studies may reflect some of these underlying differences.

Two-thirds of included studies were deemed to be at moderate or high risk of bias and this may have contributed further to variability in estimates. Although we found no evidence for statistically significant differences according to study quality, CIs and PIs were narrower for studies at low risk of bias. This suggests that we might have more confidence in pooled prevalence estimates of 8.0% for self-reported PTSD symptoms and 3.8% for clinically diagnosed PTSD generated from analyses limited to low risk of bias studies only.

There were a number of notable outliers. Ayu *et al* assessed PTSD prevalence among pregnant women in Indonesia and reported a PTSD prevalence of 41%. Almost half of participants included in this study were adolescents, who may face greater risks compared with adult perinatal populations.^29^ Türkmen *et al* assessed PTSD among postnatal women in the Republic of Türkiye and found rates of 60%, 53% and 42% at 1, 3 and 6 months post partum, respectively.^69^ The authors acknowledged that their rates could not be generalised to the wider perinatal population in Türkiye and attributed this to their study being the first to use the Turkish PTSD Short Scale.^69^ Two Brazilian studies also reported high prevalence: Angelini *et al*’s study of postnatal women found a prevalence of self-reported PTSD symptoms of 49% using the PCL-Civilian version, while Osório *et al* found a prevalence of clinically diagnosed PTSD of 30% using a diagnostic interview.^28 57^ Osório *et al* suggest that their findings might be explained by high levels of previous mental disorders, obstetric complications and infant health conditions among their community sample.^57^

### Strengths and limitations

This is the first systematic review to provide a pooled estimate of perinatal PTSD prevalence in LMICs.

The inclusion of PTSD symptoms as well as clinically diagnosed PTSD provides a comprehensive overview and recognises those who experience symptoms without meeting diagnostic criteria. The focus on community-based rather than high-risk samples means pooled estimates are likely to represent the general population of pregnant and postnatal women across LMICs. The review incorporates studies conducted before, during and after the COVID-19 pandemic and provides updated estimates. The majority of included studies were conducted after 2017 when the previous review was published. An extensive grey literature search which identified 12 additional studies and the inclusion of publications in any language represent further strengths.

There are also a number of limitations to the review. Statistical heterogeneity between studies was high (I^2^>90%) and remained significant in subgroup analyses. Meta-analyses of prevalence often have high I^2^ values but this is not always discriminative.^78^ Potential sources of heterogeneity were further explored using meta-regression and by assessing the impact on pooled estimates of using fixed-effects rather than random-effects meta-analysis. We recognise that excluding high-risk samples may be considered a limitation and that our results may underestimate the true burden of perinatal PTSD in LMICs. However, our rationale for focusing on general perinatal samples was that we wanted to understand the burden within—and the resulting resource implications for—general perinatal settings. High-risk groups exposed to specific trigger events are likely to follow different care pathways. Nevertheless, future studies should consider exploring higher risk groups. The lack of data on stressor events is a further limitation. We were unable to explore the difference in estimates between cohort studies and cross-sectional studies. This is important as the former allow more detailed insights into the incidence and evolution of PTSD and how prevalence might change over the perinatal period. Finally, the review was limited by a lack of studies from low-income (as opposed to middle-income) countries. Over two-thirds of included studies were from six countries, limiting the generalisability of findings across LMICs.

### Implications for research and clinical practice

Currently, identification and treatment of women with perinatal PTSD is low in LMICs.^79^ Screening for PTSD symptoms during routine antenatal and postpartum appointments has been recommended to facilitate detection, though feasibility and effectiveness of such screening programmes will vary between settings and should be assessed according to local prevalence and existing mental health resources.^9^ Perinatal mental healthcare programmes including psychoeducational interventions that are integrated within antenatal and postpartum healthcare services have been shown to be effective.^980^,^82^ Research into perinatal PTSD in LMICs has increased as evidenced by the number of studies conducted within the last 5 years, yet significant gaps remain. Notably, studies from low-income (as opposed to middle-income) countries and certain geographical regions including East and Central Asia and North Africa are lacking. Reliable prevalence estimates from these settings are vital to better understand the burden and to inform health service planning.

## Conclusion

Perinatal PTSD represents a significant burden in LMIC, with approximately 1 in 10 women experiencing PTSD symptoms and 1 in 20 women experiencing clinically diagnosed PTSD. Our review focused on community-based samples and our pooled estimates are therefore likely to reflect the burden among general perinatal populations across LMIC. Our prevalence estimates of clinically diagnosed PTSD in LMICs are similar to prevalence reported previously. Despite recent increased research interest in this area, the evidence base remains limited in many geographical regions. Studies from low-income countries are urgently needed in order to assess the burden in these settings.

## supplementary material

10.1136/bmjph-2023-000215 online supplemental file 1

## Data Availability

All data relevant to the study are included in the article or uploaded as supplementary information.
