# Supplementary material for: Prevalence of perinatal post-traumatic stress disorder (PTSD) in low-income and middle-income countries: a systematic review and meta-analysis
Source: BMJ Public Health. 2024 May 2;2(1):e000215. doi: 10.1136/bmjph-2023-000215 (PMC11812746; doi:10.1136/bmjph-2023-000215)

Contents

	Title	Page
**SI1**	Search strategy for databases	2
**SI2**	Search terms for grey literature search	8
**SI3**	PRISMA Checklist	9
**SI4**	Adapted Joanna Briggs Institute Checklist for Studies Reporting Prevalence Data	12
**SI5**	Table of study characteristics	
**SI6**	Quality scores of included studies	13
**SI7**	Subgroup analyses	15
**SI8**	Fixed-effects meta-analyses	17
**SI9**	Meta-regression analyses	18
**SI10**	Publication bias	19

Supplementary Information

SI1. Search strategy for databases

A. Search strategy for MEDLINE

1	exp perinatal care/ or postnatal care/ or prenatal care/
2	peripartum period/ or postpartum period/ or exp pregnancy trimesters/ or pregnancy/ or gravidity/ or parity/ or parturition/ or Pregnant Women/
3	Maternal Health/
4	(prenat* or pre-nat* or antepart* or ante-part* or pregnan* or birth or childbirth or maternal or mother or natal or perinatal or peri-natal or peripartum or peri-partum or postnatal or post-natal or postpartum or post-partum or intrapartum or intra-partum or puerperal or puerperium or trimester or antenat* or ante-nat* or postbirth or post-birth).ti,ab,kw.
5	1 OR 2 OR 3 OR 4
6	stress disorders, traumatic/ or stress disorders, post-traumatic/ or stress disorders, traumatic, acute/
7	PTSD.mp. [mp=title, abstract, original title, name of substance word, subject heading word, floating sub-heading word, keyword heading word, organism supplementary concept word, protocol supplementary concept word, rare disease supplementary concept word, unique identifier, synonyms]
8	PTSS.mp.
9	traumatic stress.mp.
10	traumatic delivery.mp.
11	obstetric trauma*.m.p.
12	(birth* adj2 trauma*).mp.
13	(posttraumatic or post-traumatic or ptsd).mp.
14	6 OR 7 OR 8 OR 9 OR 10 OR 11 OR 12 OR 13
15	Developing Countries.sh,kf.
16	(Africa or Asia or Caribbean or West Indies or South America or Latin America or Central America).hw,kf,ti,ab,cp,in.
17	(Afghanistan or Angola or Armenia or Armenian or Bangladesh or Benin or Bhutan or Bolivia or Burkina Faso or Burkina Fasso or Burundi or Cambodia or Central African Republic or Chad or Comoros or Congo or Cote d'Ivoire or Ivory Coast or Djibouti or Egypt or El Salvador or Eritrea or Ethiopia or Gambia or Gaza or Georgia or Ghana or Guatemala or Guinea or Guam or Haiti or Honduras or India or Indonesia or Kenya or Kiribati or Korea or Kosovo or Kyrgyzstan or Lao PDR or Lesotho or Liberia or Madagascar or Malawi or Mali or Mauritania or Moldova or Mongolia or Morocco or Mozambique or Myanmar or Myanma or Nepal or Nicaragua or Niger or Nigeria or Pakistan or Paraguay or Philippines or Philipines or Phillipines or Phillippines or Rwanda or Ruanda or Sao Tome or Senegal or Sri Lanka or Solomon Islands or Somalia or Sudan or Swaziland or Tajikistan or Tanzania or Timor-Leste or Tokelau or Togo or Tuvalu or Uganda or Ukraine or Uzbekistan or Vanuatu or Vietnam or Viet Nam or West Bank or Yemen or Zambia or Zimbabwe).hw,kf,ti,ab,cp,in.
18	((developing or less* developed or under developed or underdeveloped or middle income or low* income or underserved or under served or deprived or poor*) adj (countr* or nation? or population? or world)).ti,ab.
19	((developing or less* developed or under developed or underdeveloped or middle income or low* income) adj (economy or economies)).ti,ab.
20	(low* adj (gdp or gnp or gross domestic or gross national)).ti,ab.
21	(low adj3 middle adj3 countr*).ti,ab.
22	(lmic or lmics or third world or lami countr*).ti,ab.
23	transitional countr*.ti,ab.
24	((high burden or high-burden or countdown) adj countr*).ti,ab.
25	15 OR 16 OR 17 OR 18 OR 19 OR 20 OR 21 OR 22 OR 23 OR 24
26	5 AND 14 AND 25

B. Search strategy for Embase

1	exp perinatal care/ or postnatal care/ or prenatal care/
2	peripartum period/ or postpartum period/ or exp pregnancy trimesters/ or pregnancy/ or gravidity/ or parity/ or parturition/
3	Maternal Health/

4	(prenat* or pre-nat* or antepart* or pregnan* or birth or childbirth or maternal or mother or natal or perinatal or peri-natal or peripartum or peri-partum or postnatal or post-natal or postpartum or post-partum or puerperal or puerperium or trimester or antenat* or postbirth or post-birth).ti,ab,kw.
5	1 or 2 or 3 or 4
6	stress disorders, traumatic/ or stress disorders, post-traumatic/ or stress disorders, traumatic, acute/
7	PTSD.mp. [mp=title, abstract, heading word, drug trade name, original title, device manufacturer, drug manufacturer, device trade name, keyword heading word, floating subheading word, candidate term word]
8	PTSS.mp.
9	post-traumatic stress disorder.mp.
10	traumatic stress.mp.
11	traumatic delivery.mp.
12	birth trauma.mp OR birth traumas.mp OR traumatic birth.mp OR traumatic births.mp
13	posttraumatic stress.mp.
14	post-traumatic stress.mp.
15	(posttraumatic or post-traumatic or ptsd).mp.
16	6 or 7 or 8 or 9 or 10 or 11 or 12 or 13 or 14 or 15
17	developing country/
18	(Africa or Asia or Caribbean or West Indies or South America or Latin America or Central America).hw,kw,ti,ab,cp,in.
19	(Afghanistan or Angola or Armenia or Armenian or Bangladesh or Benin or Bhutan or Bolivia or Burkina Faso or Burkina Fasso or Burundi or Cambodia or Central African Republic or Chad or Comoros or Congo or Cote d'Ivoire or Ivory Coast or Djibouti or Egypt or El Salvador or Eritrea or Ethiopia or Gambia or Gaza or Georgia or Ghana or Guatemala or Guinea or Guam or Haiti or Honduras or India or Indonesia or Kenya or Kiribati or Korea or Kosovo or Kyrgyzstan or Lao PDR or Lesotho or Liberia or Madagascar or Malawi or Mali or Mauritania or Moldova or Mongolia or Morocco or Mozambique or Myanmar or Myanma or Nepal or Nicaragua or Niger or Nigeria or Pakistan or Paraguay or Philippines or Philipines or Phillipines or Phillippines or Rwanda or Ruanda or Sao Tome or Senegal or Sri Lanka or Solomon Islands or Somalia or Sudan or Swaziland or Tajikistan or Tanzania or Timor-Leste or Tokelau or Togo or Tuvalu or Uganda or Ukraine or Uzbekistan or Vanuatu or Vietnam or Viet Nam or West Bank or Yemen or Zambia or Zimbabwe).hw,kw,ti,ab,cp,in.
20	((developing or less* developed or under developed or underdeveloped or middle income or low* income) adj (economy or economies)).ti,ab.
21	(low* adj (gdp or gnp or gross domestic or gross national)).ti,ab.
22	(low adj3 middle adj3 countr*).ti,ab.
23	(lmic or lmics or third world or lami countr*).ti,ab.
24	transitional countr*.ti,ab.
25	((high burden or high-burden or countdown) adj countr*).ti,ab.
26	17 or 18 or 19 or 20 or 21 or 22 or 23 or 24 or 25
27	5 and 16 and 26

C. Search strategy for PsycINFO

1	exp Birth/ or exp Perinatal Period/ or exp Prenatal Care/ or exp Pregnancy/
2	exp Postnatal Period/
3	(prenat* or pre-nat* or antepart* or ante-part* or pregnan* or birth or childbirth or maternal or mother or natal or perinatal or peri-natal or peripartum or peri-partum or postnatal or post-natal or postpartum or post-partum or intrapartum or intra-partum or puerperal or puerperium or trimester or antenat* or ante-nat* or postbirth or post-birth).ab,hw,id.
4	1 or 2 or 3
5	"stress and trauma related disorders"/ or acute stress disorder/ or exp posttraumatic stress disorder/ or exp posttraumatic stress/
6	PTSD.mp.
7	PTSS.mp.

8	traumatic stress.mp.
9	traumatic delivery.mp.
10	obstetric trauma*.mp.
11	(birth* adj2 trauma*).mp. [mp=title, abstract, heading word, table of contents, key concepts, original title, tests & measures, mesh word]
12	(posttraumatic or post-traumatic or ptsd).mp. [mp=title, abstract, heading word, table of contents, key concepts, original title, tests & measures, mesh word]
13	4 or 5 or 6 or 7 or 8 or 9 or 10 or 11 or 12
14	exp Developing Countries/
15	(Africa or Asia or Caribbean or West Indies or South America or Latin America or Central America).af.
16	(Afghanistan or Angola or Armenia or Armenian or Bangladesh or Benin or Bhutan or Bolivia or Burkina Faso or Burkina Fasso or Burundi or Cambodia or Central African Republic or Chad or Comoros or Congo or Cote d'Ivoire or Ivory Coast or Djibouti or Egypt or El Salvador or Eritrea or Ethiopia or Gambia or Gaza or Georgia or Ghana or Guatemala or Guinea or Guam or Haiti or Honduras or India or Indonesia or Kenya or Kiribati or Korea or Kosovo or Kyrgyzstan or Lao PDR or Lesotho or Liberia or Madagascar or Malawi or Mali or Mauritania or Moldova or Mongolia or Morocco or Mozambique or Myanmar or Myanma or Nepal or Nicaragua or Niger or Nigeria or Pakistan or Paraguay or Philippines or Philipines or Phillipines or Phillippines or Rwanda or Ruanda or Sao Tome or Senegal or Sri Lanka or Solomon Islands or Somalia or Sudan or Swaziland or Tajikistan or Tanzania or Timor-Leste or Tokelau or Togo or Tuvalu or Uganda or Ukraine or Uzbekistan or Vanuatu or Vietnam or Viet Nam or West Bank or Yemen or Zambia or Zimbabwe).af.
17	((developing or less* developed or under developed or underdeveloped or middle income or low* income or underserved or under served or deprived or poor*) adj (countr* or nation? or population? or world)).ti,ab.
18	((developing or less* developed or under developed or underdeveloped or middle income or low* income) adj (economy or economies)).ti,ab.
19	(low* adj (gdp or gnp or gross domestic or gross national)).ti,ab.
20	(low adj3 middle adj3 countr*).ti,ab.
21	(lmic or lmics or third world or lami countr*).ti,ab.
22	transitional countr*.ti,ab.
23	((high burden or high-burden or countdown) adj countr*).ti,ab.
24	13 or 14 or 15 or 16 or 17 or 18 or 19 or 20 or 21 or 22 or 23
25	4 and 13 and 24

D. Search strategy for Scopus

1	TITLE-ABS-KEY (prenat* OR pre-nat* OR antepart* OR ante-part* OR pregnan* OR birth OR childbirth OR maternal OR mother OR natal OR perinatal OR peri-natal OR peripartum OR peri-partum OR postnatal OR post-natal OR postpartum OR post-partum OR intrapartum OR intra-partum OR puerperal OR puerperium OR trimester OR antenat* OR ante-nat* OR postbirth OR post-birth)
2	TITLE-ABS-KEY (ptsd OR ptss OR "traumatic stress" OR "traumatic delivery" OR "obstetric trauma*" OR (birth* W/2 trauma*) OR (posttraumatic OR post-traumatic OR ptsd))
3	KEY ("developing countries")
4	KEY (africa OR asia OR caribbean OR "west indies" OR "south america" OR "latin america" OR "central america")
5	TITLE-ABS (afghanistan OR angola OR armenia OR armenian OR bangladesh OR benin OR bhutan OR bolivia OR "burkina faso" OR "burkina fasso" OR burundi OR cambodia OR "central african republic" OR chad OR comoros OR congo OR "cote d'ivoire" OR "ivory coast" OR djibouti OR egypt OR "el salvador" OR eritrea OR ethiopia OR gambia OR gaza OR georgia OR ghana OR guatemala OR guinea OR guam OR haiti OR honduras OR india OR indonesia OR kenya OR kiribati OR korea OR kosovo OR kyrgyzstan OR "lao pdr" OR lesotho OR liberia OR madagascar OR malawi OR mali OR mauritania OR moldova OR mongolia OR morocco OR mozambique OR myanmar OR myanma OR nepal OR nicaragua OR niger OR nigeria OR pakistan OR paraguay OR philippines OR philipines OR phillipines OR phillippines OR rwanda OR ruanda OR "sao tome" OR senegal OR "sri lanka" OR "solomon islands" OR somalia OR sudan OR swaziland OR tajikistan OR tanzania OR "timor-leste" OR tokelau OR togo OR tuvalu OR uganda OR

	ukraine OR uzbekistan OR vanuatu OR vietnam OR "viet nam" OR "west bank" OR yemen OR zambia OR zimbabwe)
6	TITLE-ABS ((developing OR "less* developed" OR "under developed" OR underdeveloped OR "middle income" OR "low* income" OR underserved OR "under served" OR deprived OR poor*) PRE/1 (countr* OR nation? OR population? OR world))
7	TITLE-ABS ((developing OR "less* developed" OR "under developed" OR underdeveloped OR "middle income" OR "low* income") PRE/1 (economy OR economies))
8	TITLE-ABS (low* PRE/1 (gdp OR gnp OR "gross domestic" OR "gross national"))
9	TITLE-ABS (low W/3 middle W/ 3 countr*)
10	TITLE-ABS (lmic OR lmics OR "third world" OR "lami countr*")
11	TITLE-ABS (“Transitional countr*”)
12	TITLE-ABS ("high burden" OR high-burden OR countdown) PRE/1 countr*
13	3 OR 4 OR 5 OR 6 OR 7 OR 8 OR 9 OR 10 OR 11 OR 12
14	1 AND 2 AND 13

E. Search strategy for Web of Science

1	TS=((prenat* OR pre-nat* OR antepart* OR ante-part* OR pregnan* OR birth OR childbirth OR maternal OR mother OR natal OR perinatal OR peri-natal OR peripartum OR peri-partum OR postnatal OR post-natal OR postpartum OR post-partum OR intrapartum OR intra-partum OR puerperal OR puerperium OR trimester OR antenat* OR ante-nat* OR postbirth OR post-birth))
2	TS=(PTSD)
3	TS=(PTSS)
4	TS=("traumatic stress")
5	TS=("traumatic delivery")
6	TS=("obstetric trauma*")
7	TS=(birth NEAR/1 trauma*)
8	TS=(posttraumatic OR post-traumatic OR ptsd)
9	#6 OR 7 OR 8 OR 9 OR 10 OR 11 OR 12
10	TS=((africa OR asia OR caribbean OR "west indies" OR "south america" OR "latin america" OR "central america"))
11	TS=((afghanistan OR angola OR armenia OR armenian OR bangladesh OR benin OR bhutan OR bolivia OR "burkina faso" OR "burkina fasso" OR burundi OR cambodia OR "central african republic" OR chad OR comoros OR congo OR "cote d'ivoire" OR "ivory coast" OR djibouti OR egypt OR "el salvador" OR eritrea OR ethiopia OR gambia OR gaza OR georgia OR ghana OR guatemala OR guinea OR guam OR haiti OR honduras OR india OR indonesia))
12	TS=((kenya OR kiribati OR korea OR kosovo OR kyrgyzstan OR "lao pdr" OR lesotho OR liberia OR madagascar OR malawi OR mali OR mauritania OR moldova OR mongolia OR morocco OR mozambique OR myanmar OR myanma OR nepal OR nicaragua OR niger OR nigeria OR pakistan OR paraguay OR philippines OR philipines OR phillipines OR phillippines))
13	TS=(((rwanda OR ruanda OR "sao tome" OR senegal OR "sri lanka" OR "solomon islands" OR somalia OR sudan OR swaziland OR tajikistan OR tanzania OR "timor-leste" OR tokelau OR togo OR tuvalu OR uganda OR ukraine OR uzbekistan OR vanuatu OR vietnam OR "viet nam" OR "west bank" OR yemen OR zambia OR zimbabwe)))
14	TS=(((developing OR "less* developed" OR "under developed" OR underdeveloped OR "middle income" OR "low* income" OR underserved OR "under served" OR deprived OR poor*) NEAR/0 (countr* OR nation? OR population? OR world)))
15	TS=((developing OR "less* developed" OR "under developed" OR underdeveloped OR "middle income" OR "low* income") NEAR/0 (economy OR economies))
16	TS= (low* NEAR/0 (gdp OR gnp OR "gross domestic" OR "gross national"))
17	TS=((low NEAR/3 middle NEAR/3 countr*))
18	TS=((lmic OR lmics OR "third world" OR "lami countr*"))
19	TS=(“Transitional countr*”)
20	TS=(("high burden" OR high-burden OR countdown) NEAR/0 countr*)
21	14 OR 15 OR 16 OR 17 OR 18 OR 19 OR 20 OR 21 OR 22 OR 23 OR 24

22	5 AND 13 AND 25

F. Search strategy for Global Health

1	Postpartum period/ or parturition/
2	Pregnancy/ or birth/ or pregnant women/ or puerperium/
3	Postpartum interval/ or prenatal period/ or prepartum period/
4	(prenat* or pre-nat* or antepart* or ante-part* or pregnan* or birth or childbirth or maternal or mother or natal or perinatal or peri-natal or peripartum or peri-partum or postnatal or post-natal or postpartum or post-partum or intrapartum or intra-partum or puerperal or puerperium or trimester or antenat* or ante-nat* or postbirth or post-birth).mp. [mp=abstract, title, original title, broad terms, heading words, identifiers, cabicodes]
5	1 or 2 or 3 or 4 or 5 or 6 ~~or 7 or 8 or 9~~
6	post-traumatic stress disorder/
7	PTSD.mp.
8	PTSS.mp.
9	traumatic stress.mp.
10	traumatic delivery.mp.
11	obstetric trauma*.mp.
12	(birth* adj2 trauma*).mp. [mp=abstract, title, original title, broad terms, heading words, identifiers, cabicodes]
13	(posttraumatic or post-traumatic or ptsd).mp. [mp=abstract, title, original title, broad terms, heading words, identifiers, cabicodes]
14	8 OR 9 OR 10 OR 11 OR 12 OR 13 OR 14 OR 15
15	Developing countries/
16	(Africa or Asia or Caribbean or West Indies or South America or Latin America or Central America).ab,cp,hw,in,ti.
17	(Afghanistan or Angola or Armenia or Armenian or Bangladesh or Benin or Bhutan or Bolivia or Burkina Faso or Burkina Fasso or Burundi or Cambodia or Central African Republic or Chad or Comoros or Congo or Cote d'Ivoire or Ivory Coast or Djibouti or Egypt or El Salvador or Eritrea or Ethiopia or Gambia or Gaza or Georgia or Ghana or Guatemala or Guinea or Guam or Haiti or Honduras or India or Indonesia or Kenya or Kiribati or Korea or Kosovo or Kyrgyzstan or Lao PDR or Lesotho or Liberia or Madagascar or Malawi or Mali or Mauritania or Moldova or Mongolia or Morocco or Mozambique or Myanmar or Myanma or Nepal or Nicaragua or Niger or Nigeria or Pakistan or Paraguay or Philippines or Philipines or Phillipines or Phillippines or Rwanda or Ruanda or Sao Tome or Senegal or Sri Lanka or Solomon Islands or Somalia or Sudan or Swaziland or Tajikistan or Tanzania or Timor-Leste or Tokelau or Togo or Tuvalu or Uganda or Ukraine or Uzbekistan or Vanuatu or Vietnam or Viet Nam or West Bank or Yemen or Zambia or Zimbabwe).ab,cp,hw,in,ti.
18	((developing or less* developed or under developed or underdeveloped or middle income or low* income or underserved or under served or deprived or poor*) adj (countr* or nation? or population? or world)).ab,ti.
19	((developing or less* developed or under developed or underdeveloped or middle income or low* income) adj (economy or economies)).ab,ti.
20	(low adj3 middle adj3 countr*).ab,ti.
21	(lmic or lmics or third world or lami countr*).ab,ti.
22	"transitional countr*".ab,ti.
23	((high burden or high-burden or countdown) adj countr*).ab,ti.
24	17 or 18 or 19 or 20 or 21 or 22 or 23 or 24 or 25
25	7 AND 16 AND 26

G. Search strategy for Global Index Medicus

1	(tw:((prenat* OR pre-nat* OR antepart* OR ante-part* OR pregnan* OR birth OR childbirth OR maternal OR mother OR natal OR perinatal OR peri-natal OR peripartum OR peri-partum OR postnatal OR post-natal OR postpartum OR post-partum OR intrapartum OR intra-partum OR puerperal OR puerperium OR trimester OR antenat* OR ante-nat* OR postbirth OR post-birth)))))

2	(tw:(ptsd)) OR (tw:(ptss)) OR (tw:("traumatic stress")) OR (tw:("traumatic delivery")) OR (tw:("obstetric trauma*")) OR (tw:("birth trauma" OR birth traumas OR traumatic birth OR traumatic births)) OR (tw:((posttraumatic OR post-traumatic OR ptsd))))))
3	1 AND 2

SI2. Search terms for grey literature search

**Perinatal**	Maternal; perinatal; antenatal; postnatal; pregnancy; childbirth
**PTSD**	Post-traumatic stress disorder; PTSD
**LMIC**	LMIC; low income country; middle income country

SI3. PRISMA Checklist

Section and Topic	Item #	Checklist item	Location where item is reported
**TITLE**			
Title	1	Identify the report as a systematic review.	1
**ABSTRACT**			
Abstract	2	See the PRISMA 2020 for Abstracts checklist.	1
**INTRODUCTION**			
Rationale	3	Describe the rationale for the review in the context of existing knowledge.	2-3
Objectives	4	Provide an explicit statement of the objective(s) or question(s) the review addresses.	2
**METHODS**			
Eligibility criteria	5	Specify the inclusion and exclusion criteria for the review and how studies were grouped for the syntheses.	2-3
Information sources	6	Specify all databases, registers, websites, organisations, reference lists and other sources searched or consulted to identify studies. Specify the date when each source was last searched or consulted.	2
Search strategy	7	Present the full search strategies for all databases, registers and websites, including any filters and limits used.	SI2
Selection process	8	Specify the methods used to decide whether a study met the inclusion criteria of the review, including how many reviewers screened each record and each report retrieved, whether they worked independently, and if applicable, details of automation tools used in the process.	2-3
Data collection process	9	Specify the methods used to collect data from reports, including how many reviewers collected data from each report, whether they worked independently, any processes for obtaining or confirming data from study investigators, and if applicable, details of automation tools used in the process.	2-3
Data items	10a	List and define all outcomes for which data were sought. Specify whether all results that were compatible with each outcome domain in each study were sought (e.g. for all measures, time points, analyses), and if not, the methods used to decide which results to collect.	3
	10b	List and define all other variables for which data were sought (e.g. participant and intervention characteristics, funding sources). Describe any assumptions made about any missing or unclear information.	3
Study risk of bias assessment	11	Specify the methods used to assess risk of bias in the included studies, including details of the tool(s) used, how many reviewers assessed each study and whether they worked independently, and if applicable, details of automation tools used in the process.	3, SI4
Effect measures	12	Specify for each outcome the effect measure(s) (e.g. risk ratio, mean difference) used in the synthesis or presentation of results.	3
Synthesis methods	13a	Describe the processes used to decide which studies were eligible for each synthesis (e.g. tabulating the study intervention characteristics and comparing against the planned groups for each synthesis (item #5)).	3
	13b	Describe any methods required to prepare the data for presentation or synthesis, such as handling of missing summary statistics, or data conversions.	3
	13c	Describe any methods used to tabulate or visually display results of individual studies and syntheses.	3
	13d	Describe any methods used to synthesize results and provide a rationale for the choice(s). If meta-analysis was performed, describe the model(s), method(s) to identify the presence and extent of statistical heterogeneity, and software package(s) used.	3
	13e	Describe any methods used to explore possible causes of heterogeneity among study results (e.g. subgroup analysis, meta-regression).	3
	13f	Describe any sensitivity analyses conducted to assess robustness of the synthesized results.	3

Section and Topic	Item #	Checklist item	Location where item is reported
Reporting bias assessment	14	Describe any methods used to assess risk of bias due to missing results in a synthesis (arising from reporting biases).	3
Certainty assessment	15	Describe any methods used to assess certainty (or confidence) in the body of evidence for an outcome.	3
**RESULTS**			
Study selection	16a	Describe the results of the search and selection process, from the number of records identified in the search to the number of studies included in the review, ideally using a flow diagram.	Figure 1
	16b	Cite studies that might appear to meet the inclusion criteria, but which were excluded, and explain why they were excluded.	Figure 1
Study characteristics	17	Cite each included study and present its characteristics.	3-4, SI5
Risk of bias in studies	18	Present assessments of risk of bias for each included study.	4, SI5, SI6
Results of individual studies	19	For all outcomes, present, for each study: (a) summary statistics for each group (where appropriate) and (b) an effect estimate and its precision (e.g. confidence/credible interval), ideally using structured tables or plots.	4, 5, Table 1, Figure 2
Results of syntheses	20a	For each synthesis, briefly summarise the characteristics and risk of bias among contributing studies.	4, 5, SI5
	20b	Present results of all statistical syntheses conducted. If meta-analysis was done, present for each the summary estimate and its precision (e.g. confidence/credible interval) and measures of statistical heterogeneity. If comparing groups, describe the direction of the effect.	4, 5, Table 1, Figure 2
	20c	Present results of all investigations of possible causes of heterogeneity among study results.	Table 1, Figure 2
	20d	Present results of all sensitivity analyses conducted to assess the robustness of the synthesized results.	5, SI7
Reporting biases	21	Present assessments of risk of bias due to missing results (arising from reporting biases) for each synthesis assessed.	4, 5
Certainty of evidence	22	Present assessments of certainty (or confidence) in the body of evidence for each outcome assessed.	4, 5, Table 1
**DISCUSSION**			
Discussion	23a	Provide a general interpretation of the results in the context of other evidence.	5
	23b	Discuss any limitations of the evidence included in the review.	8
	23c	Discuss any limitations of the review processes used.	8
	23d	Discuss implications of the results for practice, policy, and future research.	7
**OTHER INFORMATION**			
Registration and protocol	24a	Provide registration information for the review, including register name and registration number, or state that the review was not registered.	2
	24b	Indicate where the review protocol can be accessed, or state that a protocol was not prepared.	2
	24c	Describe and explain any amendments to information provided at registration or in the protocol.	3
Support	25	Describe sources of financial or non-financial support for the review, and the role of the funders or sponsors in the review.	8
Competing interests	26	Declare any competing interests of review authors.	8
Availability of data, code and other materials	27	Report which of the following are publicly available and where they can be found: template data collection forms; data extracted from included studies; data used for all analyses; analytic code; any other materials used in the review.	9

From: Page MJ, McKenzie JE, Bossuyt PM, Boutron I, Hoffmann TC, Mulrow CD, et al. The PRISMA 2020 statement: an updated guideline for reporting systematic reviews. BMJ 2021;372:n71.

SI4. Adapted Joanna Briggs Institute Checklist for Studies Reporting Prevalence Data

	Yes	No	Unclear	N/A
1. Was the sample frame appropriate to address the target population?	□	□	□	□
2. Were study participants sampled in an appropriate way?	□	□	□	□
3. Was the sample size adequate?	□	□	□	□
4. Were the study subjects and the setting described in detail?	□	□	□	□
5. Was the data analysis conducted with sufficient coverage of the identified sample?	□	□	□	□
6. Were valid methods used for the identification of the condition?	□	□	□	□
7. Was the condition measured in a standard, reliable way for all participants?	□	□	□	□
8. Was there appropriate statistical analysis?	□	□	□	□
9. Was the response rate adequate, and if not, was the low response rate managed appropriately?	□	□	□	□

SI5. Table of study characteristics of included studies

Reference<br>(Publication language)	Country	Setting<br>(Urban/Rural)	Study design	Timing of assessment	Outcome	Measure<br>(cut-off)	Participants	Prevalence (%)	Risk of bias
Adewuya 2006a<br>(English)	Nigeria	Community<br>(Urban)	Cross-sectional	PN (6w)	PTSD<br>diagnosis	MINI	876	5.9	Low
Adewuya 2006b<br>(English)	Nigeria	Community<br>(Urban)	Case-control	AN (3rd)	PTSD<br>diagnosis	MINI	172	0.6	Low
Alemu 2020<br>(English)	Tanzania	Hospital<br>(Urban-rural)	Cohort[ǂ]	PN (3m)<br>PN (6m)<br>PN (12m)	PTSD	HTQ-16 (≥2.5)	PN (3m): 146<br>PN (6m): 113<br>PN (12m): 114	PN (3m): 0<br>PN (6m): 0<br>PN (12m): 0	Moderate
Andretto 2010<br>(Portuguese)	Brazil	Hospital<br>(Urban)	Cross-sectional	PN (6w)	PTSD	PCL-C (Cluster)	135	10.0	Low
Angelini 2018[a]<br>(English)	Brazil	Hospital<br>(Urban)	Cohort[ǂ]	PN (6–12m)	PTSD	PCL-C (≥40)	55	41.8	Moderate
Ayu 2019*<br>(English)	Indonesia	Community<br>(Urban)	Cross-sectional	AN (any)	PTSD	PSS (>13)	214	40.7	High
Busari 2010*<br>(English)	Nigeria	Both<br>(NR)	Cross-sectional	AN (any)	PTSD<br>diagnosis	DIS	682	5.6	Moderate
Dikmen-Yildiz 2017a*[#]<br>*PACT*<br>(English)	Türkiye	Hospital<br>(Urban)	Cohort	AN (3rd)<br>PN (4–6w)<br>PN (6m)	PTSD	PDS (Cluster)	950	AN (3rd):5.8<br>PN (4–6w): 11.9<br>PN (6m): 9.7	Low
Dikmen-Yildiz 2017b*<br>*PACT*<br>(English)	Türkiye	Hospital<br>(Urban)	Cohort	AN (3rd)<br>PN (4–6w)<br>PN (6m)	PTSD	PDS (Cluster)	950	AN (3rd): 5.8<br>PN (4–6w): 11.9<br>PN (6m): 9.7	Low
Ennazk 2021<br>(English)	Morocco	Community<br>(NR)	Cross-sectional	PN (0–6m)	PTSD	PPQ-C (≥19)	302	28.8	High
Fadzil 2013<br>(English)	Malaysia	Hospital<br>(NR)	Cross-sectional	AN (any)	PTSD<br>diagnosis	MINI	175	0.0	Moderate
Farias 2013<br>(English)	Brazil	Community<br>(Urban)	Cohort	AN (1st)	PTSD<br>diagnosis	MINI	239	1.7	Moderate
Ferreira 2020[a,#]<br>(English)	Brazil	Hospital<br>(Urban)	Cohort	PN (6m–5y)	PTSD	PCL-C (≥50)	412	27.2	High
Fonseca-Machado 2015<br>(English)	Brazil	Hospital<br>(Urban)	Cross-sectional	AN (3rd)	PTSD	PCL-C (Cluster)	358	17	Low

Friedman 2017[#]<br>*PrOMIS*<br>(English)	Peru	Hospital<br>(Urban)	Cross-sectional	AN (any)	PTSD	PCL-C (≥26)	2922	37.4	Low
Gan 2019<br>(Chinese)	China	Hospital<br>(Urban)	Cross-sectional	PN (1w–6m)	PTSD	PPQ-C (≥19)	1160	16.2	Moderate
Gankanda 2021<br>(English)	Sri Lanka	Community<br>(Semi-urban)	Cohort	PN (1m)<br>PN (2m)<br>PN (6m)	PTSD	PSS-SR (>13)	PN (1m): 225 PN<br>(2m): 214 PN<br>(6m): 211	PN (1m): 2.7<br>PN (2m): 0.9<br>PN (6m): 0.5	Low
Gelaye 2020<br>*PrOMIS*[#]<br>(English)	Peru	Hospital<br>(Urban)	Cohort	AN (1st)	PTSD	PCL-C (≥26)	4194	34.2	Low
Ghorbani 2014<br>(English)	Iran	Community<br>(Urban)	Cross-sectional	PN (2m)	PTSD	PSS (Cluster)	82	1.2	High
Gökçe 2016[#]<br>(English)	Türkiye	Community<br>(Urban)	Cohort	AN (<20w)	PTSD	IES-R (≥30)	242	NR	Low
Koen 2017[#]<br>*DCHS*<br>(English)	South<br>Africa	Community<br>(Semi-urban)	Cohort	PN (6m)	PTSD<br>diagnosis	MINI	111	20.0	Moderate
Koen 2021[#]<br>*DCHS*<br>(English)	South<br>Africa	Community<br>(Semi-urban)	Cohort	AN (any)	PTSD	mPSS (Cluster)	271	30.0	High
Kumar 2016<br>(English)	India	Hospital<br>(Urban)	Cross-sectional	PN (0–2w)	PTSD<br>diagnosis	MINI	152	0.0	High
Kumar 2019<br>(English)	India	Hospital<br>(NR)	Cross-sectional	PN (first<br>follow-up)	PTSD<br>diagnosis	MINI	200	0.0	High
Liu 2021<br>(English)	China	Hospital<br>(Urban)	Cross-sectional	PN (6–8w)	PTSD	PPQ (≥19)	1136	6.1	Moderate
MacGinty 2020[#]<br>*DCHS*<br>(English)	South<br>Africa	Community<br>(Semi-urban)	Cohort	AN (3rd)	PTSD	mPSS (Cluster)	961	13.1	Low
Mahenge 2013[b]<br>(English)	Tanzania	Hospital<br>(Urban)	Cross-sectional	AN (any)	PTSD	PDS (≥11)	1180	12.7	Low
Mahenge 2015[b,#]<br>(English)	Tanzania	Hospital<br>(Urban)	Cross-sectional	AN (any)	PTSD	PDS (≥11)	1180	4.9	Moderate
Mahmoodi 2016<br>(English)	Iran	Hospital<br>(Urban)	Cohort	PN (6-8w)	PTSD	PSS (Cluster)	240	6.2	Moderate

Malaju 2022a [c,*,#]<br>(English)	Ethiopia	Hospital<br>(Semi-urban)	Cohort [ǂ]	PN (6w)<br>PN (12w)<br>PN (18w)	PTSD	PCL-5 (≥33)	775	PN (6w): 8.3<br>PN (12w): 5.8<br>PN (18w): 2.8	Low
Malaju 2022b [*,c]<br>(English)	Ethiopia	Community<br>(Semi-urban)	Cohort [ǂ]	PN (6w)<br>PN (12w)<br>PN (18w)	PTSD	PCL-5 (≥33)	775	PN (6w): 8.3<br>PN (12w): 5.8<br>PN (18w): 2.8	Low
Maré 2021<br>*DCHS*<br>(English)	South Africa	Community<br>(Semi-urban)	Cohort	AN (2nd & 3rd)<br>PN (6m)	PTSD diagnosis	MINI	AN: 347<br>PN: 522	AN: 17.7<br>PN: 15.2	High
Milosavljevic 2016<br>(English)	Serbia	Hospital<br>(Urban)	Cohort	PN (1m)<br>PN (2m)<br>PN (3m)	PTSD	CAPS (≥19)	126	PN (1m): 11.9<br>PN (2m): 5.9<br>PN (3m): 1.3	High
Osório 2021<br>(English)	Brazil	Online<br>(NR)	Validation	PN (0–12m)	PTSD diagnosis	SCID	60	30.0	High
Rashid 2020<br>(English)	Pakistan	Hospital<br>(Semi-urban)	Case-control	AN (any)	PTSD diagnosis	MINI	225	5.8	Low
Sanchez 2017<br>*PrOMIS*<br>(English)	Peru	Hospital<br>(Urban)	Cohort	AN (1st & 2nd)	PTSD	PCL-C (≥44)	2928	6.2	Low
Sanchez 2020<br>*PrOMIS*<br>(English)	Peru	Hospital<br>(Urban)	Cohort	AN (1st & 2nd)	PTSD	PCL-C (≥26)	2051	30.9	High
Shaban 2013<br>(English)	Iran	Hospital<br>(Urban)	Cross-sectional	PN (6–8w)	PTSD	PSS (Cluster)	600	17.2	Moderate
Shen 2020 [*,#]<br>*PrOMIS*<br>(English)	Peru	Hospital<br>(Urban)	Cross-sectional	AN (1st & 2nd)	PTSD	PCL-C (median)	?	?	Moderate
Shiva 2021<br>(English)	India	Community & hospital<br>(Urban)	Cross-sectional	PN (2–6w)	PTSD	PCL-C (≥44)	95	7.4	High
Sileo 2019<br>(English)	Liberia	Community<br>(Urban)	Cross-sectional	AN (2nd)	PTSD	PCL-C (NR)	191	0.0	Moderate
Silove 2015<br>(English)	Timor-Leste	Community<br>(Mixed)	Cross-sectional	AN (2nd)<br>PN (3–6m)	PTSD	HTQ (≥2.0)	AN: 257<br>PN: 170	AN: 7.4<br>PN: 12.4	Low
Silva 2010<br>(English)	Brazil	Community<br>(Urban)	Cross-sectional	AN (2nd & 3rd)	PTSD diagnosis	MINI	260	3.1	High

Spies 2009<br>(English)	South Africa	Hospital<br>(Urban)	Validation	AN (1st & 2nd)	PTSD diagnosis	SCID	129	3.1	High
Tavares 2012<br>(English)	Brazil	Community<br>(Urban)	Cross-sectional	PN (1–3m)	PTSD diagnosis	MINI	919	3.6	High
Tol 2018<br>*DILI*<br>(English)	Timor-Leste	Community<br>(Mixed)	Cohort	AN (2nd)	PTSD	HTQ-16 (≥2.0)	1627	5.7	High
Türkmen 2020<br>(English)	Türkiye	Hospital<br>(NR)	Cohort	PN (4w)<br>PN (3m)<br>PN (6m)	PTSD	Turkish PTSD-Short Scale (24)	102	PN (4w): 59.8<br>PN (3m): 52.9<br>PN (6m): 42.2	Moderate
van Heyningen 2017<br>(English)	South Africa	Community<br>(Urban)	Cross-sectional	AN (any)	PTSD diagnosis	MINI	376	11.0	Moderate
Yakupova 2022<br>(English)	Russia	Online	Cross-sectional	PN (0-12m)	PTSD	CBTS (NR)	611	15.1	High
Yang 2016#<br>*PrOMIS*<br>(English)	Peru	Hospital<br>(Urban)	Cross-sectional	AN (1st)	PTSD	PCL-C (≥26)	2928	37.4	Low
Zambaldi 2011<br>(English)	Brazil	Hospital<br>(Urban)	Cross-sectional	PN (2–26w)	PTSD diagnosis	MINI	400	5.3	High

Abbreviations
1st first trimester of pregnancy; *2nd* second trimester of pregnancy; *3rd* third trimester of pregnancy; *AN* antenatal; *CAPS* Clinician-Administered PTSD Scale; *CBTS* City Birth Trauma Scale; *DCHS* Drakenstein Child Health Study; *DILI* Desenvolvimentu Isin-d'iak Labarik No Inan Birth Cohort; *DIS* Diagnostic Interview Schedule; *HTQ-16* Harvard Trauma Questionnaire; *IES-R* Impact of Events Scale – Revised; *m* months; *MINI* Mini International Psychiatric Interview; *mPSS* modified PTSD Symptom Scale*; NR* not reported; *PACT* Pregnancy and Childbirth in Turkey*; PCL-5* PTSD Checklist for DSM-5; *PCL-C* PTSD Checklist – Civilian Version; *PDS* Posttraumatic Diagnostic Scale; *PN* postnatal; *PSS* PTSD Symptom Scale; *PSS-SR* PTSD Symptom Scale Self Report; *PPQ*-Perinatal PTSD Questionnaire; *PPQ-C* Perinatal PTSD Questionnaire – Chinese Version; *PrOMIS* Pregnancy Outcomes, Maternal and Infant Cohort Study; *SCID* Structured Clinical Interview for DSM-IV Disorders; *w* weeks.

Notes
*****Additional information received from authors
[#] Not included in meta-analysis
[ǂ] Data used in meta-analysis represents non-exposed participants only
[a,b,c] Linked publications reporting data from the same participants

SI6. Quality scores of included studies

Authors	1. Sample frame appropriate to address target population?	2. Study participants sampled appropriately?	3. Sample size adequate?	4. Subjects and setting described in detail?	5. Data analysis: sufficient coverage of identified sample?	6. Valid methods to identify condition?	7. Condition measured in standard, reliable way for all participants?	8. Appropriate statistical analysis?	9. Response rate adequate? If not, low response rate managed appropriately?	Overall risk of bias*
Adewuya 2006a	Yes	Yes	No	Yes	Yes	Yes	Yes	Yes	Yes	**Low**
Adewuya 2006b	Yes	Yes	No	Yes	Yes	Yes	Yes	Yes	Yes	**Low**
Alemu 2020	Yes	Yes	Unclear	Yes	Yes	Yes	Yes	Unclear	Yes	**Moderate**
Andretto 2010	Yes	Unclear	Yes	Yes	Yes	Yes	Yes	Yes	Yes	**Low**
Angelini 2018	Unclear	Yes	Yes	Yes	Unclear	Yes	Yes	Yes	Yes	**Moderate**
Ayu 2019	Unclear	Yes	No	Yes	Yes	Yes	Unclear	Unclear	Unclear	**High**
Busari 2010	Yes	Yes	No	Yes	Yes	Yes	Unclear	Yes	Yes	**Moderate**
Dikmen-Yildiz 2017a	Yes	Yes	Yes	Yes	Yes	Yes	Unclear	Yes	Yes	**Low**
Dikmen-Yildiz 2017b	Yes	Yes	Yes	Yes	Yes	Yes	Unclear	Yes	Yes	**Low**
Ennazk 2021	Unclear	Unclear	No	No	Yes	Yes	Yes	Yes	Unclear	**High**
Fadzil 2013	Yes	Unclear	No	Yes	Yes	Yes	Unclear	Yes	Yes	**Moderate**
Farias 2013	Yes	No	No	Yes	Unclear	Yes	Yes	Yes	Yes	**Moderate**
Ferreira 2020	Unclear	Unclear	Yes	Unclear	No	Yes	Yes	Yes	Yes	**High**
Fonseca-Machado 2015	Yes	Yes	Yes	Yes	Yes	Yes	Yes	Yes	Yes	**Low**
Friedman 2017	Yes	Yes	Yes	Yes	Yes	Yes	Yes	Yes	Yes	**Low**
Gan 2019	Yes	Yes	No	Unclear	Yes	Yes	Yes	Yes	Yes	**Moderate**
Gankanda 2021	Yes	Unclear	Yes	Yes	Yes	Yes	Yes	Yes	Yes	**Low**
Gelaye 2020	Yes	Yes	Yes	Yes	Unclear	Yes	Yes	Yes	Yes	**Low**
Ghorbani 2014	Yes	Unclear	No	Yes	Unclear	Yes	Unclear	Yes	Yes	**High**
Gökçe 2016	Yes	Yes	No	Yes	Yes	Yes	Yes	Yes	Yes	**Low**
Koen 2017	Yes	Yes	Yes	Yes	Unclear	Yes	Yes	Yes	Unclear	**Moderate**
Koen 2021	Unclear	Unclear	No	Yes	Unclear	Yes	Yes	Yes	Yes	**High**
Kumar 2016	Yes	Yes	Unclear	Yes	Yes	Yes	Unclear	Unclear	Unclear	**High**
Kumar 2019	Yes	Unclear	Unclear	Unclear	Unclear	Yes	Unclear	Unclear	Unclear	**High**
Liu 2021	Yes	No	No	Yes	Yes	Yes	Yes	Yes	Yes	**Moderate**
MacGinty 2020	Yes	Yes	Yes	Yes	Yes	Yes	Yes	Yes	Yes	**Low**
Mahenge 2013	Yes	Yes	No	Yes	Yes	Yes	Yes	Yes	Yes	**Low**
Mahenge 2015	Yes	Yes	No	Yes	Unclear	Yes	Yes	Yes	Yes	**Moderate**
Mahmoodi 2016	Yes	Yes	Yes	Yes	Yes	Yes	Unclear	Yes	Unclear	**Moderate**
Malaju 2022a	Yes	Yes	Yes	Yes	Yes	Yes	Yes	Yes	Yes	**Low**
Malaju 2022b	Yes	Yes	Yes	Yes	Yes	Yes	Yes	Yes	Yes	**Low**
Maré 2021	Yes	Unclear	No	Yes	Unclear	Yes	Yes	Yes	Unclear	**High**
Milosavljevic 2016	Unclear	Yes	No	Yes	Yes	Yes	Unclear	Yes	Unclear	**High**

Osório 2021	Unclear	Yes	Unclear	Yes	Unclear	Yes	Yes	Yes	No	**High**
Rashid 2020	Yes	Yes	Yes	Yes	Yes	Yes	Yes	Yes	Yes	**Low**
Sanchez 2017	Yes	Yes	Yes	Yes	Yes	Yes	Yes	Yes	Yes	**Low**
Sanchez 2020	Yes	Yes	Yes	Yes	Unclear	Yes	Yes	Yes	Yes	**Low**
Shaban 2013	Yes	Yes	No	Yes	Yes	Yes	Yes	Yes	Unclear	**Moderate**
Shen 2020	Yes	Yes	No	Yes	Unclear	Yes	Yes	No	Yes	**Moderate**
Shiva 2021	Unclear	No	No	Yes	Yes	Yes	Unclear	Yes	Unclear	**High**
Sileo 2019	Yes	Yes	No	Yes	Unclear	Yes	Yes	Unclear	Yes	**Moderate**
Silove 2015	Yes	Yes	No	Yes	Yes	Yes	Yes	Yes	Yes	**Low**
Silva 2010	Yes	Unclear	No	Yes	Yes	Yes	Yes	Unclear	Unclear	**High**
Spies 2009	Yes	Yes	No	No	Unclear	Yes	Yes	Yes	No	**High**
Tavares 2012	Yes	Unclear	No	No	Yes	Yes	Unclear	Yes	Yes	**High**
Tol 2018	Yes	Yes	Unclear	Yes	Unclear	Yes	Unclear	Unclear	Yes	**High**
Türkmen 2020	Yes	Unclear	Yes	Yes	Unclear	Yes	Unclear	Yes	Yes	**Moderate**
van Heyningen 2017	Yes	Unclear	No	Yes	Yes	Yes	Yes	Yes	Unclear	**Moderate**
Yakupova 2022	Unclear	Unclear	No	Unclear	Unclear	Yes	Unclear	Yes	Unclear	**High**
Yang 2016	Yes	Yes	Yes	Yes	Yes	Yes	Yes	Yes	Yes	**Low**
Zambaldi 2011	Yes	No	No	No	Yes	Yes	Unclear	Yes	Yes	**High**

Scored using Joanna Briggs Institute (JBI) Critical Appraisal Checklist for Studies Reporting Prevalence (Munn Z, Moola S, Lisy K, Riitano D, Tufanaru C. Methodological guidance for systematic reviews of observational epidemiological studies reporting prevalence and incidence data. Int J Evid Based Healthcare. 2015;13(3):147–153).

*Overall risk of bias: each study was categorised as being at low (8 or 9 quality criteria met), moderate (6 or 7 quality criteria met) or high (5 or fewer quality criteria met) risk of bias.

SI7. Subgroup analyses

Note: For each subgroup analysis, self-reported PTSD is shown on the left and clinically-diagnosed PTSD disorder is shown on the right.

Antenatal vs. postnatal (right: self-reported PTSD symptoms; left: clinically-diagnosed PTSD)

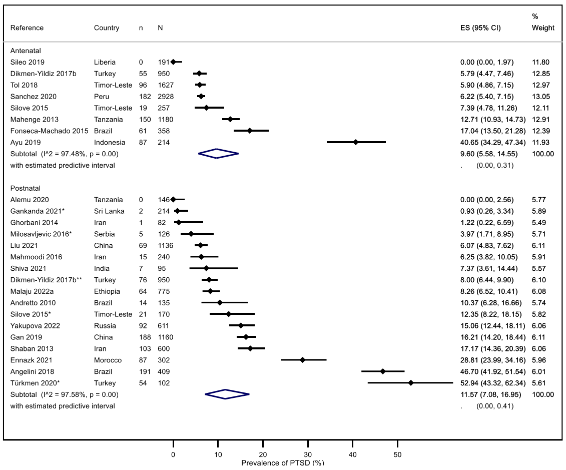

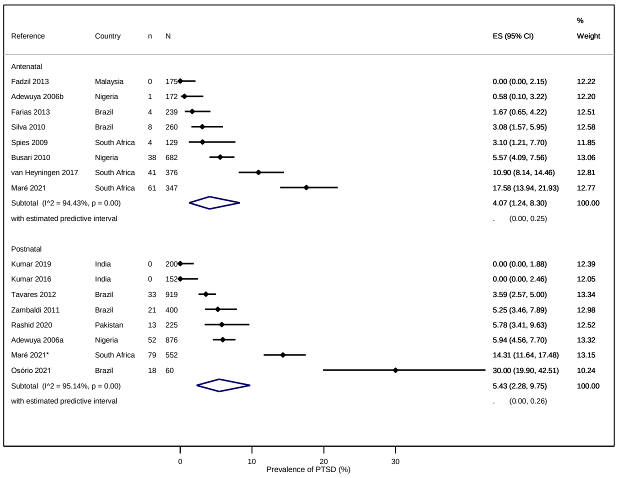

Early (0-1m) vs. late (1-12m) postnatal (right: self-reported PTSD symptoms; left: clinically-diagnosed PTSD)

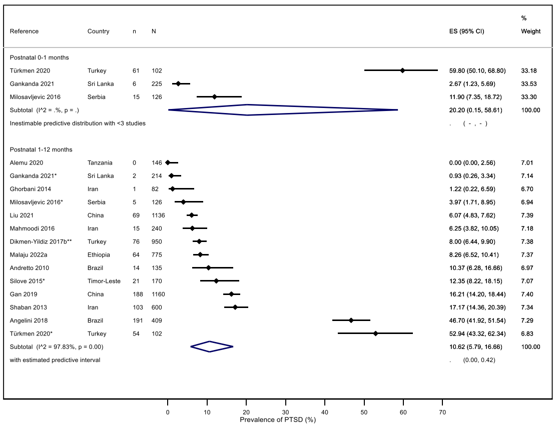

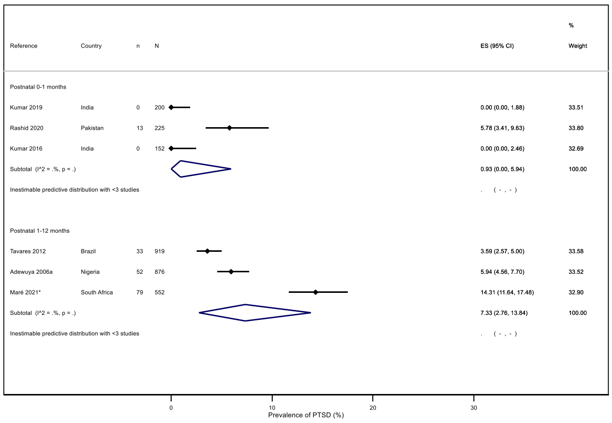

Geographical region (right: self-reported PTSD symptoms; left: clinically-diagnosed PTSD)

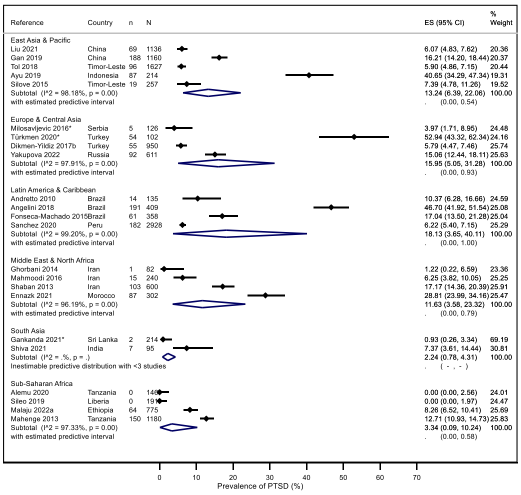

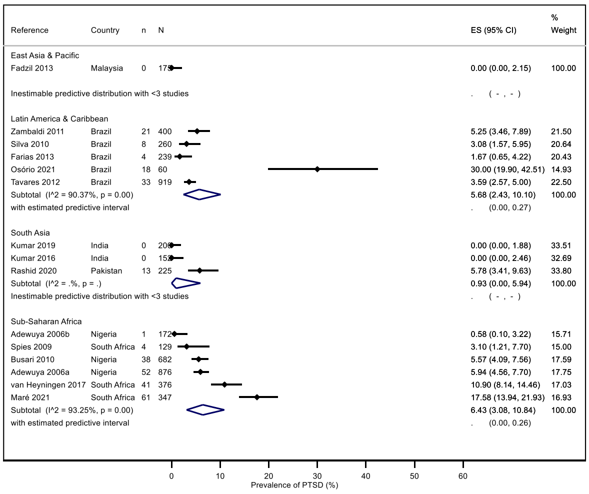

Community vs. hospital setting (right: self-reported PTSD symptoms; left: clinically-diagnosed PTSD)

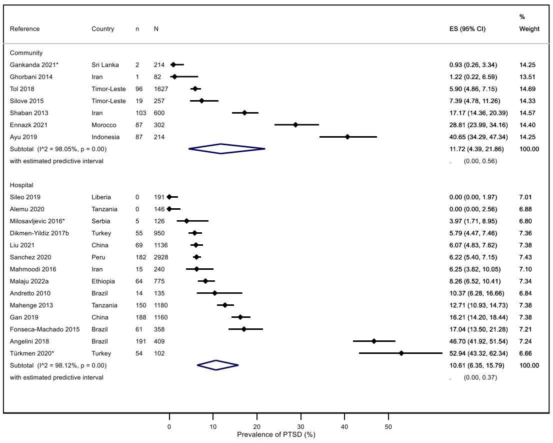

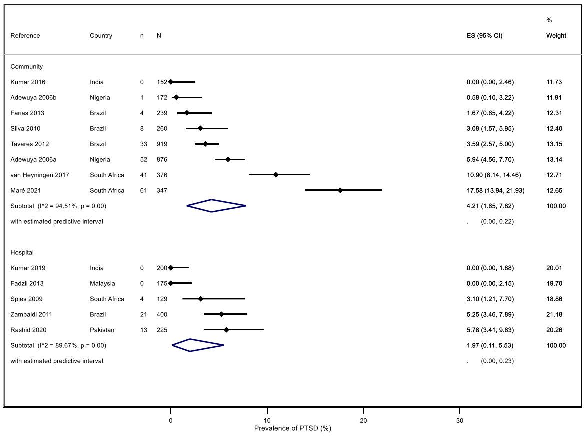

Risk of bias (right: self-reported PTSD symptoms; left: clinically-diagnosed PTSD)

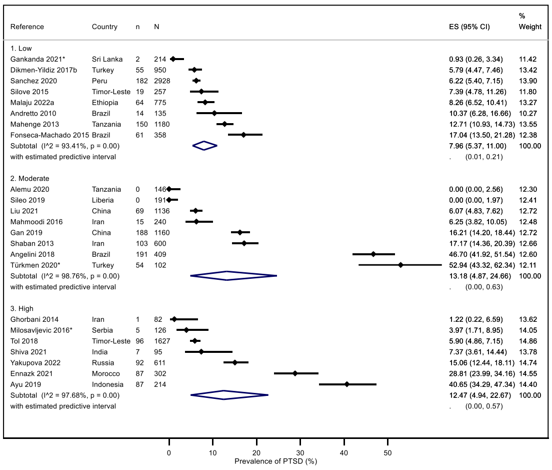

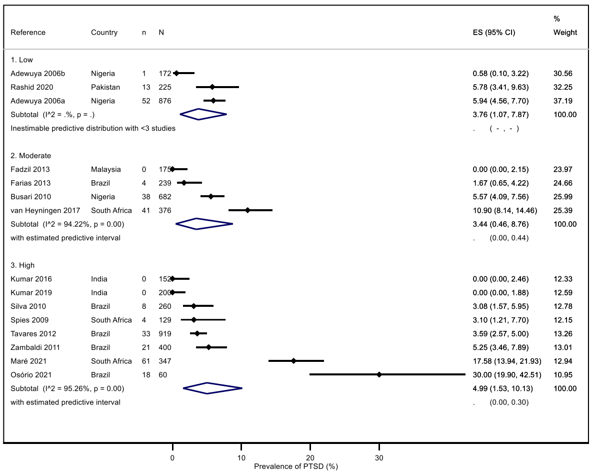

SI8. Fixed-effects meta-analyses

Pooled prevalence of PTSD by perinatal stage, geographical region, setting and study quality (fixed-effects meta-analysis)

	PTSD symptoms		PTSD disorder	
	Studies (sample)	Pooled prevalence (95% CI)	Studies (sample)	Pooled prevalence (95% CI)
**All studies**	23 (13,838)	9.8 (9.3–10.3)	15 (5212)	4.6 (4.1–5.2)
**Perinatal stage**				
Antenatal	8 (7705)	7.7 (7.1–8.3)	8 (2380)	5.4 (4.5–6.4)
Postnatal	17 (7253)	12.0 (11.3–12.8)	8 (3384)	5.3 (4.6–6.1)
**Postnatal stage**				
Early (0-1 m)	3 (453)	13.7 (10.7–17.1)	3 (577)	1.2 (0.4–2.3)
Late (1-12 m)	14 (6245)	11.2 (10.4–12.0)	3 (2347)	6.5 (5.5–7.5)
**Geographical region***				
East Asia & Pacific	5 (4394)	9.5 (8.7–10.4)	1 (175)	--
Europe & Central Asia	4 (1789)	10.1 (8.8–12.0)	0	--
Latin America	4 (3830)	10.1 (9.1–11.0)	5 (1878)	3.9 (3.0–4.8)
Middle East & N. Africa	4 (1224)	15.6 (13.6–17.7)	0	--
South Asia	2 (309)	2.2 (0.8–4.3)	3 (577)	1.2 (0.4–2.3)
Sub-Saharan Africa	4 (2292)	8.2 (7.1–9.3)	6 (2582)	7.0 (6.0–8.0)
**Setting***				
Community	7 (3296)	10.2 (9.2–11.3)	8 (3341)	5.1 (4.4–5.9)
Hospital	14 (9836)	9.4 (8.8–9.9)	5 (1129)	2.5 (1.6–3.5)
**Study quality***				
High	8 (6797)	10.7 (9.6–11.8)	3 (1273)	4.5 (3.7–5.3)
Moderate	8 (3984)	13.2 (12.1–14.2)	3 (1472)	4.8 (3.7–5.6)
Low	7 (3057)	7.7 (7.0–8.3)	8 (2467)	4.9 (3.7–6.1)

* Antenatal estimates and postnatal estimates combined

SI9. Meta-regression analyses

Meta-regression analyses for PTSD (random-effects analysis)

	**Self-reported PTSD symptoms**				**Clinically-diagnosed PTSD**			
	Pooled proportion (95% CI)	LR test	df	p-value	Pooled proportion (95% CI)	LR test	df	p-value
**Perinatal stage**								
Antenatal	0.08 (0.03-0.18)	0.15	1	0.70	0.03 (0.01-0.08)	0.28	1	0.67
Postnatal	0.09 (0.05-0.16)				0.04 (0.01-0.11)			
**Postnatal stage**								
Early (0-1 month)	0.15 (0.04-0.46)	0.58	1	0.45	0.01 (0.00-0.04)	3.68	1	0.06
Late (1-12 months)	0.08 (0.04-0.16)				0.07 (0.06-0.08)			
**Geographical region**								
East Asia & Pacific	0.12 (0.05-0.27)				--			
Europe & Central Asia	0.13 (0.05-0.33)				--			
Latin America	0.16 (0.06-0.38)	7.76	5	0.17	0.05 (0.02-0.14)	8.61	3	**0.03**
Middle East & N. Africa	0.10 (0.03-0.26)				--			
South Asia	0.03 (0.00-0.14)				0.01 (0.00-0.06)			
Sub-Saharan Africa	0.03 (0.01-0.09)				0.05 (0.02-0.15)			
**Setting**								
Community	0.09 (0.03-0.21)	0.01	1	0.92	0.03 (0.01-0.08)	0.79	1	0.37
Hospital	0.08 (0.04-0.15)				0.02 (0.00-0.06)			
**Study quality**								
Low	0.10 (0.04-0.24)				0.03 (0.00-0.16)			
Moderate	0.08 (0.03-0.19)	0.26	2	0.88	0.02 (0.00-0.11)	0.36	2	0.84
High	0.07 (0.03=0.16)				0.03 (0.01-0.10)			

SI10. Publication bias

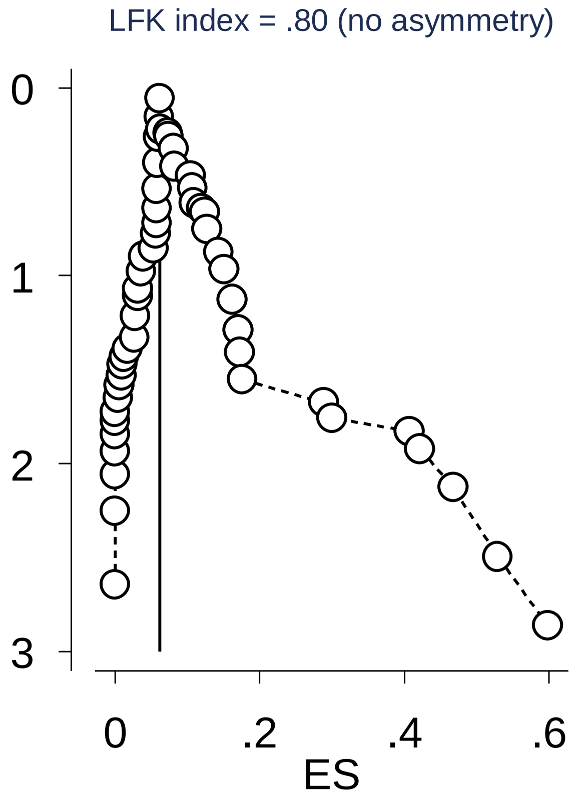

Supplement: online supplemental file 1 [file bmjph-2-1-s001.pdf]
